# Abrocitinib may improve itch and quality of life in patients with itch‐dominant atopic dermatitis

**DOI:** 10.1002/ski2.382

**Published:** 2024-05-05

**Authors:** Jonathan I. Silverberg, Jacob P. Thyssen, Irina Lazariciu, Daniela E. Myers, Erman Güler, Raj Chovatiya

**Affiliations:** ^1^ Department of Dermatology The George Washington University School of Medicine and Health Sciences Washington District of Columbia USA; ^2^ Department of Dermatology Bispebjerg Hospital University of Copenhagen Copenhagen Denmark; ^3^ Department of Biostatistics IQVIA Kirkland Quebec Canada; ^4^ Pfizer Inc. Collegeville Pennsylvania USA; ^5^ Pfizer Inc. Istanbul Turkey; ^6^ Chicago Medical School, Rosalind Franklin University of Medicine and Science North Chicago Illinois USA; ^7^ Center for Medical Dermatology and Immunology Research Chicago Illinois USA

## Abstract

**Background:**

Patients with atopic dermatitis (AD) exhibit heterogeneous clinical phenotypes, reflecting different combinations of itch and lesional severity. AD with severe itch but clear‐moderate lesions, also known as itch‐dominant AD, is a common clinical phenotype.

**Objectives:**

To evaluate abrocitinib efficacy in patients with moderate‐to‐severe AD who have itch‐dominant AD.

**Methods:**

This post hoc analysis includes pooled data from clinical trials of patients with moderate‐to‐severe AD receiving abrocitinib (100 or 200 mg) as monotherapy (phase 2b; phase 3 JADE MONO‐1 and JADE MONO‐2) or in combination with topical therapy (phase 3 JADE COMPARE). Data from the ongoing long‐term JADE EXTEND trial (data cutoff April 2020) were also evaluated. Itch‐dominant AD was defined as baseline Peak Pruritus Numerical Rating Scale (PP‐NRS) score of 7−10 and Investigator's Global Assessment of 0−3 or Eczema Area and Severity Index of 0‒21. Assessments included a ≥4‐point improvement in PP‐NRS (PP‐NRS4), PP‐NRS score of 0 (no itch) or 1 (little itch) in patients with PP‐NRS score ≥2 at baseline, ≥4‐point improvement from baseline in Patient‐Oriented Eczema Measure (POEM‐4), Patient Global Assessment (PtGA) of clear or almost clear, and Dermatology Life Quality Index (DLQI) score of 0 or 1 (no impact or little impact of AD on quality of life [QoL]).

**Results:**

In the pooled monotherapy trials, 37% of patients had itch‐dominant AD at baseline. As early as Week 2, more patients with itch‐dominant AD achieved PP‐NRS4 with abrocitinib 100 mg (35%) and abrocitinib 200 mg (57%) versus placebo (7%); 6% and 22% versus 0%, respectively, achieved PP‐NRS 0/1. More patients achieved a PtGA of clear/almost clear at Week 12 with abrocitinib 100 mg (28%) and abrocitinib 200 mg (45%) than placebo (9%). Additionally, abrocitinib led to clinically meaningful improvements in POEM and DLQI. Most patients with itch‐dominant AD experienced itch improvement over time with abrocitinib monotherapy or with concomitant topical therapy; 86%–87% and 62%–67% of patients had no itch‐moderate itch and clear‐moderate lesions by weeks 24 and 48, respectively.

**Conclusions:**

Abrocitinib is highly efficacious in patients with itch‐dominant AD, demonstrating rapid, deep, and sustained improvements in itch and clinically meaningful improvements in patients' QoL.

**Trial Registration Numbers:**

NCT02780167; NCT03349060; NCT03575871; NCT03720470; NCT03422822.

## Graphical abstract

This analysis of pooled data from randomized clinical trials showed that abrocitinib, an oral, once‐daily, JAK‐1 selective inhibitor is efficacious in patients identified as having itch‐dominant atopic dermatitis. Treatment with abrocitinib resulted in rapid, deep, and sustained improvements in itch and clinically meaningful improvements in quality of life in this subset of patients with high unmet need. Most patients with itch‐dominant atopic dermatitis at baseline improved over the long term.

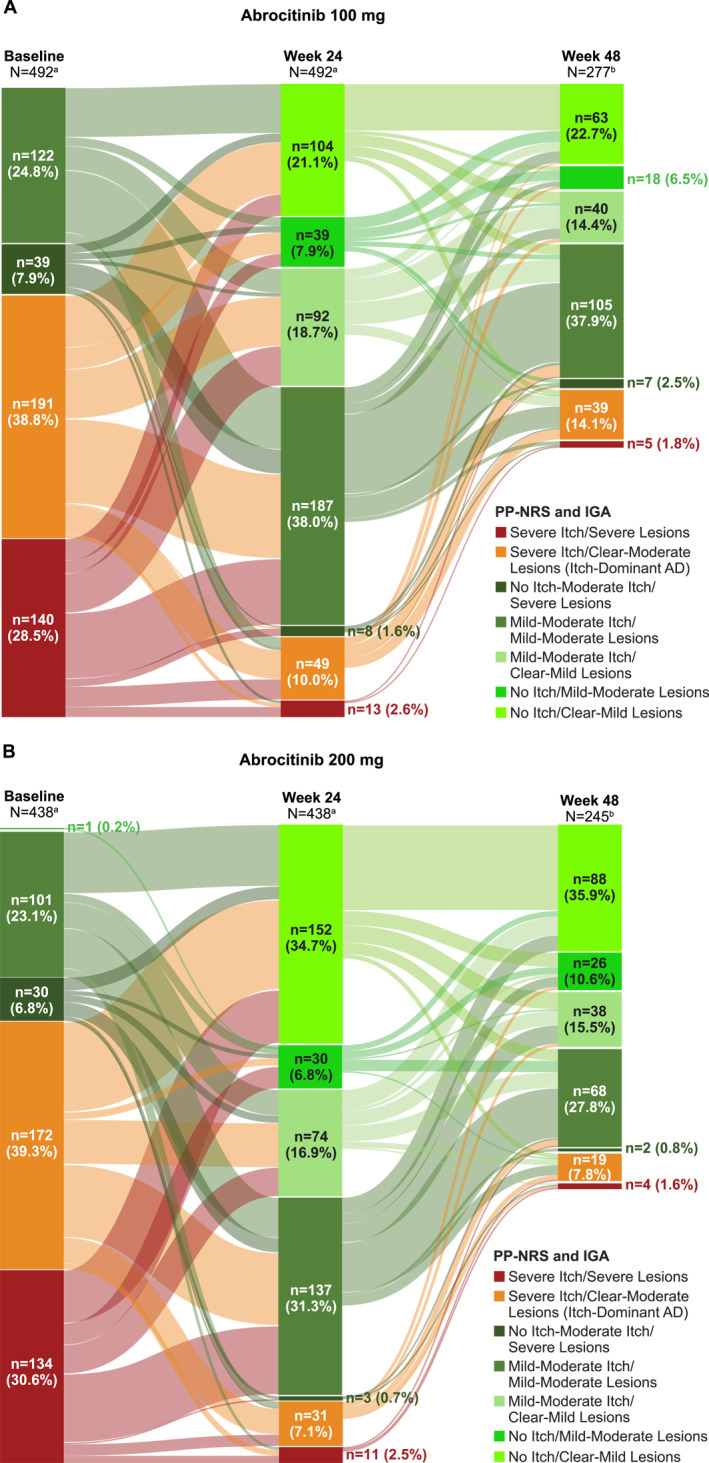

**What is already known about this topic?**

- Atopic dermatitis (AD) is a chronic inflammatory skin disorder characterised by itch and eczematous lesions
- AD presenting with severe itch but clear‐moderate lesional severity, or itch‐dominant AD, is a recently identified, yet common clinical phenotype
- Abrocitinib, an oral, once‐daily, Janus kinase 1‐selective inhibitor approved for the treatment of moderate‐to‐severe AD, has shown itch relief and skin clearance in patients in randomised clinical trials

**What does this study add?**

- Abrocitinib monotherapy provided rapid reductions in itch and disease severity and improved quality of life by Week 2 in patients with itch‐dominant AD
- Most patients with itch‐dominant AD experienced improvement over time with abrocitinib treatment; 86%–87% and 62%–67% of patients had no itch‐moderate itch and clear‐moderate lesions by weeks 24 and 48, respectively
- Abrocitinib is efficacious in patients with itch‐dominant AD

## 1 INTRODUCTION

Patients with atopic dermatitis (AD) experience variable itch and lesional severity over the course of their disease.^1^ The lesional severity, along with extent and location, contributes to the burden of AD; however, the clinical assessment of lesions alone does not capture the full patient perspective.^2^, ^3^ Itch is frequently reported by patients as the most burdensome symptom of AD^2^, ^4^; greater itch severity is associated with poor sleep and mental health, including anxiety and depression, and lower QoL.^4^, ^5^

Itch is often correlated with the extent and severity of the skin lesions, but many patients have disproportionately more itch than lesions and experience itch worsening even when active lesions are no longer visible.^6^ Indeed the correlation between itch and lesional severity has been shown to be weak to moderate at best.^7^ In a prospective observational standard‐of‐care study, approximately 25% of patients presented with severe itch but mild or moderate lesions, a clinical phenotype which is referred to as itch‐dominant AD.^6^ These patients have more disease flares and itch triggers than patients with mild itch and moderate or severe lesions and were more likely to describe their AD as severe but less likely to be categorised as such by health care providers.^6^ In a large Danish registry study of patients with AD, itch was predominantly confined to lesional skin in mild AD but also involved nonlesional skin as the severity of disease increased.^8^

The inconsistencies between itch and lesional severity may be due in part to the limited reliability of common scoring measures of lesional severity across skin tones. In clinical practice, both the SCORing AD (SCORAD) index and Eczema Area and Severity Index (EASI) vastly underestimate the severity of erythema in patients with darker complexions.^9^, ^10^ This disparity may lead to a discordance between physician‐ and patient‐reported AD severity, assessed through metrics such as Patient‐Oriented Eczema Measure (POEM) and Patient Global Assessment (PtGA). For optimal patient care and clinical management, it is important to address the heterogeneity of AD, accounting for both the subjective patient experience and objective clinical disease characteristics.

Although clinical trials for moderate‐to‐severe AD have included patients having the itch‐dominant phenotype, this subset has not been studied independently, and identification and optimal management of these patients remains a challenge. Abrocitinib is an oral, once‐daily, Janus kinase 1 (JAK1)‐selective inhibitor approved for the treatment of adults^11^, ^12^, ^13^, ^14^ and adolescents^11^, ^12^, ^13^ with moderate‐to‐severe AD. This post hoc analysis aimed to evaluate the short‐ and long‐term efficacy of abrocitinib in patients with itch‐dominant AD.

## 2 PATIENTS AND METHODS

### 2.1 Study design and treatment

In this post hoc study, data were pooled for analysis from the abrocitinib 100 mg, abrocitinib 200 mg, and placebo arms of the phase 2b (NCT02780167), and the phase 3 JADE MONO‐1 (NCT03349060) and JADE MONO‐2 trials (NCT03575871),^15^, ^16^, ^17^ and separately from the abrocitinib 100 mg and abrocitinib 200 mg, and placebo arms across the JADE MONO‐1, JADE MONO‐2, and JADE COMPARE (NCT03720470) trials that continued into JADE EXTEND, an ongoing long‐term extension study (NCT03422822; data cutoff April 2020).^18^, ^19^

Patients were ≥12 years of age (≥18 years in the phase 2b monotherapy study and phase 3 JADE COMPARE) with a diagnosis of moderate‐to‐severe AD defined as ≥10% affected body surface area; Investigator's Global Assessment (IGA) score ≥3; EASI score ≥16 (>12 in phase 2b); Peak Pruritus Numerical Rating Scale (PP‐NRS^20^; used with permission from Regeneron Pharmaceuticals, Inc. and Sanofi) score ≥4 (JADE MONO‐1, JADE MONO‐2 and JADE COMPARE only) for ≥1 year and a history of inadequate response to treatment with medicated topical therapy or had required systemic therapy. Patients who received abrocitinib treatment in the JADE MONO‐1 and JADE MONO‐2 trials for 12 weeks, and the JADE COMPARE trial for 20 weeks continued to receive the same abrocitinib dose in JADE EXTEND. Patients who received placebo in the JADE MONO‐1 and JADE MONO‐2 trials were randomly assigned to receive abrocitinib 100 or 200 mg in JADE EXTEND. Patients who received placebo in the JADE COMPARE trial were randomly assigned at Week 16 to receive abrocitinib 100 or 200 mg for up to Week 20 in the study and continued to receive the same abrocitinib dose in JADE EXTEND. All evaluated studies were approved by institutional review boards or ethics committees at each study site, and all patients provided written informed consent.

### 2.2 Assessments

Patients with moderate‐to‐severe AD identified as having itch‐dominant AD (severe itch and clear‐moderate lesions) were assessed short‐term over 12 weeks after treatment with abrocitinib (100 or 200 mg) or placebo in the JADE monotherapy pool, and long‐term up to Week 48 after treatment with abrocitinib (100 or 200 mg) in the JADE EXTEND population.

Patients with itch‐dominant AD in the monotherapy pool were defined as having a baseline PP‐NRS score of 7−10 and IGA score of 3 or EASI score of 12−21. Short‐term efficacy assessments (over 12 weeks) in this subset included the proportion of patients with itch‐dominant AD who achieved a ≥4‐point improvement in PP‐NRS (PP‐NRS4; in patients with PP‐NRS ≥4 at baseline), PP‐NRS score of 0 (no itch) or 1 (little itch) (PP‐NRS 0/1 in patients with PP‐NRS score ≥2 at baseline), ≥4‐point improvement from baseline in POEM (POEM‐4), PtGA of clear or almost clear, and Dermatology Life Quality Index (DLQI) score of 0 (no impact of AD on QoL) or 1 (little impact of AD on QoL).

Patients with itch‐dominant AD in the long‐term JADE EXTEND pooled population were defined as having a baseline PP‐NRS score of 7−10 and IGA score of 0−3 or EASI score of 0−21. Changes from baseline in itch and lesional severity were evaluated in this subset following 24‐ and 48‐week of treatment with abrocitinib. Long‐term efficacy of abrocitinib was also evaluated in subgroups of patients who had no itch‐moderate itch (PP‐NRS 0−6) and severe lesions (IGA 4 or EASI >21), no itch‐moderate itch (PP‐NRS 0−6) and clear‐moderate lesions (IGA 0−3 or EASI 0−21), and severe itch (PP‐NRS 7−10) and severe lesions (IGA 4 or EASI >21).

### 2.3 Statistical analysis

This post hoc analysis was conducted in the full analysis set, defined as all randomly assigned patients who received ≥1 dose of study medication. For binary outcomes assessed in the monotherapy pool, the 95% confidence intervals were calculated using the normal approximation of binomial proportions. Patients who permanently discontinued the study were considered as nonresponders at all visits following the discontinuation.

## 3 RESULTS

### 3.1 Demographics and baseline disease characteristics

Of 937 patients in the pooled monotherapy trials, 351 (37.5%) and 198 (21.1%) patients were categorised as having itch‐dominant AD according to baseline PP‐NRS and IGA scores and PP‐NRS and EASI scores, respectively. Baseline characteristics of the itch‐dominant subset were largely comparable with the overall population (Table 1). Severe AD according to PtGA score was reported by 57.4% and 53.1% of patients with itch‐dominant AD defined by baseline PP‐NRS and IGA scores and PP‐NRS and EASI scores, respectively, compared with 49.1% of patients in the overall pooled monotherapy population (Table 1). In the itch‐dominant AD subset defined by PP‐NRS and IGA, all patients had IGA of 3; and mean EASI ranged from 22.9 to 25.2 across the treatment arms. In the itch‐dominant AD subset defined by PP‐NRS and EASI, 78.5%–93.5% of patients across the treatment arms had IGA of 3; 6.5%–21.5% of patients had IGA of 4; and mean EASI ranged from 17.4 to 18.1 (Table 1).

**TABLE 1:** Patient demographics and baseline disease characteristics in patients with itch‐dominant AD and overall study population in the monotherapy pool.

	Itch‐dominant AD						Overall study population (monotherapy pool) *n* = 942		
	By baseline PP‐NRS and IGA scores *n* = 351			By baseline PP‐NRS and EASI scores *n* = 198					
	Placebo *n* = 78	Abrocitinib 100 mg *n* = 137	Abrocitinib 200 mg *n* = 136	Placebo *n* = 46	Abrocitinib 100 mg *n* = 73	Abrocitinib 200 mg *n* = 79	Placebo *n* = 210	Abrocitinib 100 mg *n* = 369	Abrocitinib 200 mg *n* = 363
Age, mean (SD), y	35.9 (15.6)	38.8 (15.7)	34.5 (15.8)	37.8 (16.1)	35.9 (17.1)	36.0 (16.5)	35.0 (15.0)	35.9 (15.8)	34.1 (16.4)
Male, *n* (%)	36 (46.2)	67 (48.9)	67 (49.3)	18 (39.1)	31 (42.5)	37 (46.8)	117 (55.7)	215 (58.3)	197 (54.3)
Race/ethnicity, *n* (%)									
Asian	13 (16.7)	32 (23.4)	26 (19.1)	7 (15.2)	15 (20.5)	14 (17.7)	39 (18.6)	80 (21.7)	85 (23.4)
Black/African American	9 (11.5)	11 (8.0)	14 (10.3)	5 (10.9)	6 (8.2)	9 (11.4)	22 (10.5)	31 (8.4)	30 (8.3)
White	53 (67.9)	91 (66.4)	87 (64.0)	32 (69.6)	51 (69.9)	48 (60.8)	141 (67.1)	253 (68.6)	231 (63.6)
Hispanic/Latino	3 (3.8)	6 (4.4)	2 (1.5)	2 (4.3)	6 (8.2)	3 (3.8)	11 (5.2)	14 (3.8)	12 (3.3)
Fitzpatrick skin type, *n* (%)									
I–III	48 (61.5)	86 (62.8)	94 (69.1)	32 (69.6)	48 (65.8)	49 (62.0)	136 (64.8)	237 (64.2)	249 (68.6)
IV–VI	30 (38.5)	50 (36.5)	42 (30.9)	14 (30.4)	25 (34.2)	30 (38.0)	74 (35.2)	131 (35.5)	114 (31.4)
Disease duration, median (range), y	19.8 (12.8−27.9)	22.2 (11.9−35.8)	19.1 (11.5−32.7)	18.6 (12.3−27.9)	17.2 (7.0−29.2)	18.7 (10.4−31.1)	20.8 (13.1−30.5)	21.2 (11.1−35.0)	18.9 (10.8−30.8)
Age of onset^a^, mean (SD), y	14.4 (17.7)	15.6 (19.5)	12.9 (16.2)	16.6 (18.6)	16.9 (20.0)	15.4 (17.6)	12.8 (16.5)	13.4 (17.6)	13.3 (17.9)
% BSA, mean (SD)	40.1 (19.7)	43.9 (20.5)	43.2 (21.6)	29.2 (12.0)	28.1 (10.1)	27.7 (11.4)	45.8 (22.1)	48.6 (22.5)	47.2 (23.6)
EASI score, mean (SD)	22.9 (7.9)	25.2 (8.5)	25.1 (10.6)	17.7 (2.4)	18.1 (2.2)	17.4 (2.7)	27.6 (11.8)	29.4 (12.4)	29.0 (13.4)
IGA category, *n* (%)									
Moderate (IGA 3)	78 (100.0)	137 (100.0)	136 (100.0)	43 (93.5)	59 (80.8)	62 (78.5)	132 (62.9)	228 (61.8)	231 (63.6)
Severe (IGA 4)	0	0	0	3 (6.5)	14 (19.2)	17 (21.5)	78 (37.1)	141 (38.2)	132 (36.4)
Pruritus‐NRS/PP‐NRS score^b^, mean (SD)	8.1 (1.0)	8.0 (1.0)	8.0 (0.9)	8.1 (1.1)	8.1 (1.0)	8.0 (1.0)	7.0 (1.9)	7.1 (1.9)	7.0 (1.9)
PtGA category^c^, *n* (%)									
Clear	0	0	0	0	0	0	0	1 (0.3)	0
Almost clear	1 (1.3)	0	0	0	0	0	5 (2.4)	3 (0.8)	2 (0.6)
Mild	1 (1.3)	3 (2.2)	4 (2.9)	1 (2.2)	2 (2.7)	2 (2.5)	8 (3.8)	23 (6.2)	21 (5.8)
Moderate	39 (50.0)	41 (29.9)	60 (44.1)	24 (52.2)	26 (35.6)	37 (46.8)	101 (48.1)	142 (38.5)	169 (46.6)
Severe	37 (47.4)	93 (67.9)	71 (52.2)	21 (45.7)	43 (58.9)	40 (50.6)	95 (45.2)	196 (53.1)	168 (46.3)
Comorbid asthma at baseline, *n* (%)									
Yes	28 (35.9)	46 (33.6)	42 (30.9)	18 (39.1)	24 (32.9)	27 (34.2)	73 (34.8)	131 (35.5)	115 (31.7)
No	50 (64.1)	91 (66.4)	94 (69.1)	28 (60.9)	49 (67.1)	52 (65.8)	137 (65.2)	238 (64.5)	248 (68.3)

*Note*: In the pooled monotherapy population, patients with itch‐dominant AD at baseline had severe itch (PP‐NRS 7−10) and (IGA 3 or EASI 12−21).

Abbreviations: AD, atopic dermatitis; BSA, body surface area; EASI, Eczema Area and Severity Index; IGA, Investigator's Global Assessment; NRS, numerical rating scale; PP‐NRS, Peak Pruritus Numerical Rating Scale; PP‐NRS4; improvement of ≥4 points from baseline in PP‐NRS score; QD, once daily.

^a^ Age of disease onset was self‐reported.

^b^ PP‐NRS score collected at baseline for *n* = 351 patients in the itch‐dominant AD group by IGA score, *n* = 198 in the itch‐dominant AD group by EASI score, and 937 patients in the overall study population (monotherapy pool).

^c^ PtGA was completed at baseline by 135, 137, and 78 patients with itch‐dominant AD by IGA score treated with abrocitinib 200 mg, abrocitinib 100 mg, and placebo, respectively; by 79, 71, and 46 patients with itch‐dominant AD by EASI score; and by 360, 365, and 209 patients in the overall study population (monotherapy pool).

### 3.2 Short‐term efficacy over 12 weeks in the monotherapy pool

#### 3.2.1 Itch response in patients with itch‐dominant AD

**FIGURE 1:**
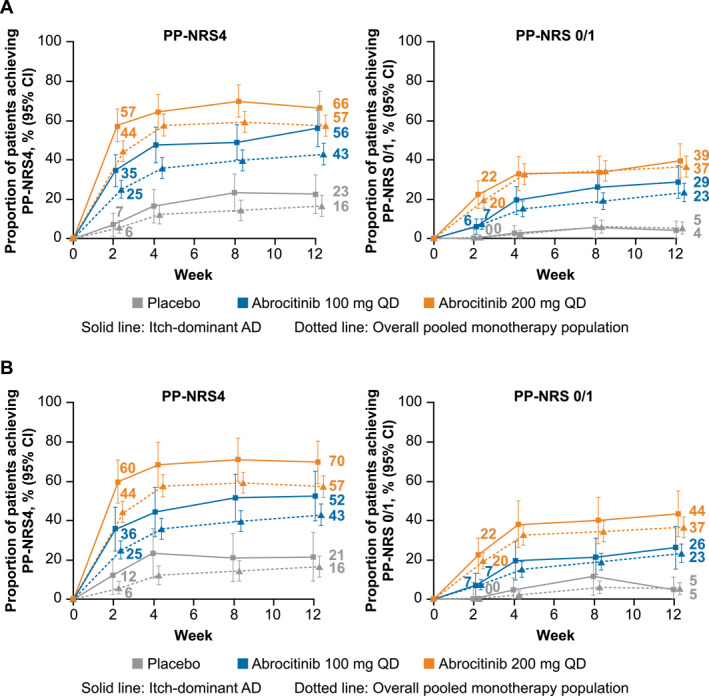
Proportion of patients with itch‐dominant AD defined by baseline (a) IGA score or (b) EASI score who achieved PP‐NRS4 and PP‐NRS 0/1 responses compared with the overall pooled monotherapy population. AD, atopic dermatitis; EASI, Eczema Area and Severity Index; IGA, Investigator's Global Assessment; PP‐NRS, Pruritus Numerical Rating Scale/Peak Pruritus Numerical Rating Scale; PP‐NRS4; improvement of ≥4 points from baseline in PP‐NRS score; QD, once daily. Pruritus Numerical Rating Scale was assessed in the phase 2b trial; Peak Pruritus Numerical Rating Scale was assessed in JADE MONO‐1 and JADE MONO‐2. PP‐NRS<2 response was assessed in patients with PP‐NRS score of ≥2 at baseline. Patients with itch‐dominant AD at baseline had severe itch (PP‐NRS 7−10) and IGA 3 or EASI 12−21.

In the itch‐dominant AD subset defined by IGA score, 46/133 patients (35%) in the abrocitinib 100 mg group and 77/134 patients (57%) in the abrocitinib 200 mg group achieved PP‐NRS4 at Week 2, compared with 5/72 patients (7%) in the placebo group (Figure 1a). At Week 12, these proportions increased to 62/111 patients (56%), 79/119 patients (66%), and 16/71 patients (23%), respectively.

In addition, the stringent response of PP‐NRS 0/1 at Week 2 was achieved by 8/133 patients (6%) with abrocitinib 100 mg and 30/134 patients (22%) with abrocitinib 200 mg, compared with 0/72 patients (0%) with placebo (Figure 1a). At Week 12, these proportions increased to 32/111 (29%), 47/119 (39%), and 3/71 (4%), respectively.

Similar results were observed in the itch‐dominant AD subset defined by EASI score (Figure 1b). The proportion of patients who achieved PP‐NRS4 and PP‐NRS 0/1 was largely comparable in the itch‐dominant AD subsets (by baseline IGA or EASI) and the overall pooled monotherapy population.

#### 3.2.2 Patient‐reported outcomes in patients with itch‐dominant AD

**FIGURE 2:**
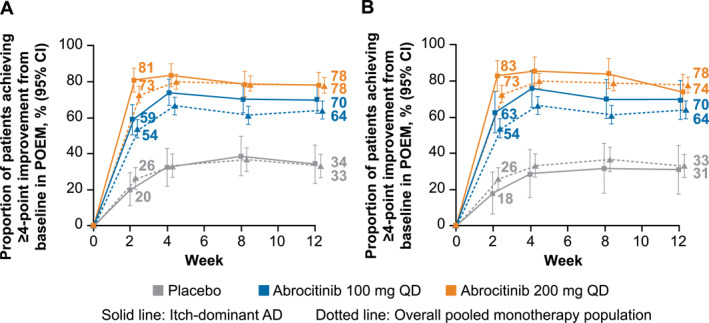
Proportion of patients with itch‐dominant AD defined by baseline (a) IGA score or (b) EASI score who achieved a ≥4‐point improvement in POEM compared with the overall pooled monotherapy population. AD, atopic dermatitis; EASI, Eczema Area and Severity Index; IGA, Investigator's Global Assessment; POEM, Patient‐Oriented Eczema Measure; PP‐NRS, Peak Pruritus Numerical Rating Scale; QD, once daily. Patients with itch‐dominant AD at baseline had severe itch (PP‐NRS 7−10) and IGA 3 or EASI 12−21.

In the subset of patients with itch‐dominant AD defined by IGA score, 79/134 patients (59%) in the abrocitinib 100 mg group and 106/131 patients (81%) in the abrocitinib 200 mg group achieved POEM‐4 at Week 2, compared with 15/74 patients (20%) in the placebo group (Figure 2a). These proportions were maintained through Week 12, with 94/134 patients (70%), 103/132 patients (78%), and 26/76 patients (34%), respectively, achieving POEM‐4. Similar results were observed in the subset with itch‐dominant AD defined by EASI score (Figure 2b). POEM score improvements in the itch‐dominant subsets (defined by baseline IGA or EASI score) were consistent with those in the overall pooled monotherapy population.

**FIGURE 3:**
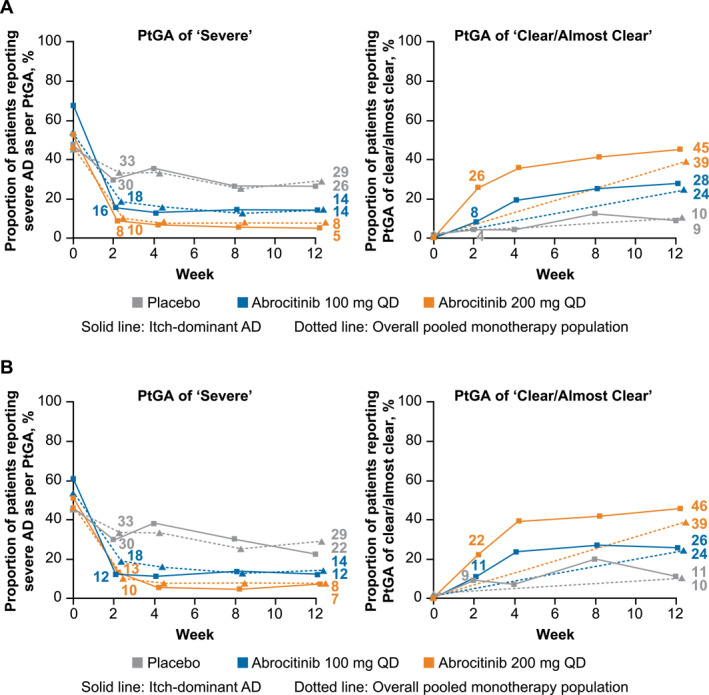
Proportion of patients with itch‐dominant AD defined by baseline (a) IGA score or (b) EASI score who had PtGA of severe disease and PtGA of clear or almost clear compared with the overall pooled monotherapy population. AD, atopic dermatitis; EASI, Eczema Area and Severity Index; IGA, Investigator's Global Assessment; PP‐NRS, Peak Pruritus Numerical Rating Scale; PtGA, Patient Global Assessment; QD, once daily. Patients with itch‐dominant AD at baseline had severe itch (PP‐NRS 7−10) and IGA 3 or EASI 12−21.

The proportions of patients with itch‐dominant AD defined by IGA score who reported severe disease based on PtGA score decreased from 68% at baseline to 16% at Week 2 after treatment with abrocitinib 100 mg, from 53% to 8% with abrocitinib 200 mg, and from 47% to 30% with placebo (Figure 3a). These improvements in disease severity were sustained through Week 12. Similar improvements were observed in the subset with itch‐dominant AD defined by EASI score (Figure 3b) and the overall pooled monotherapy population.

At Week 12 after treatment, the stringent threshold of PtGA of clear or almost clear was achieved in 35/126 patients (28%) with abrocitinib 100 mg and in 55/122 patients (45%) with abrocitinib 200 mg compared with 5/57 patients (9%) in the placebo group in the itch‐dominant AD subset defined by IGA score (Figure 3a). Similar results were observed in the itch‐dominant subset defined by EASI score (Figure 3b) and the overall pooled monotherapy population.

**FIGURE 4:**
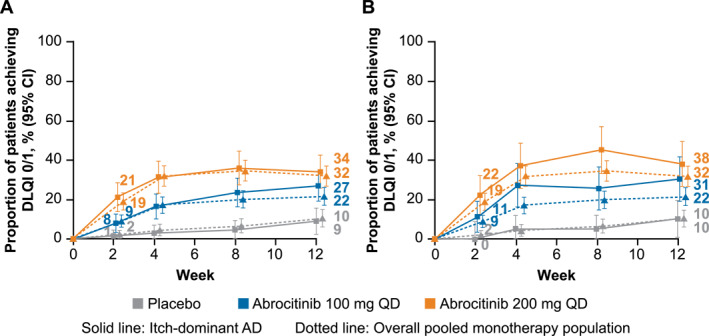
Proportion of patients with itch‐dominant AD defined by baseline (a) IGA score or (b) EASI score who achieved DLQI 0/1 compared with overall pooled monotherapy population. AD, atopic dermatitis; DLQI, Dermatology Life Quality Index; EASI, Eczema Area and Severity Index; IGA, Investigator's Global Assessment; PP‐NRS, Peak Pruritus Numerical Rating Scale; QD, once daily. Analysis includes patients with a DLQI score ≥2 at baseline. Patients with itch‐dominant AD at baseline had severe itch (PP‐NRS 7−10) and IGA 3 or EASI 12−21.

In the itch‐dominant AD subset defined by IGA score, DLQI 0/1 (little to no impact of AD on QoL) was reported in 34/127 adult patients (27%) in the abrocitinib 100 mg group and 41/120 patients (34%) in the abrocitinib 200 mg group compared with 6/67 patients (9%) in the placebo group at Week 12 after treatment (Figure 4a). Similar results were observed in the itch‐dominant AD subset defined by baseline EASI score (Figure 4b). DLQI improvements in the itch‐dominant subsets (defined by baseline IGA or EASI score) were comparable with the overall pooled monotherapy population.

### 3.3 Changes in itch and lesional severity over 48 weeks

Of 1116 patients who enrolled in the long‐term JADE EXTEND study, 930 patients (492 [abrocitinib 100 mg]; 438 [abrocitinib 200 mg]) had available data at baseline and Week 24. Of those, 39% had itch‐dominant AD at baseline in each of the abrocitinib treatment groups (191/492 patients [abrocitinib 100 mg; Figure 5a]; 172/438 patients [abrocitinib 200 mg; Figure 5b]) as assessed by PP‐NRS and IGA scores. At Week 24 of the long‐term extension study, these proportions decreased to 10% (49/492) in the abrocitinib 100 mg group and 7% (31/438) in the abrocitinib 200 mg group. Of patients with available data at Week 24 who reached Week 48 in the ongoing EXTEND study, 14% (39/277) in the abrocitinib 100 mg group (Figure 5a) and 8% (19/245) in the abrocitinib 200 mg group (Figure 5b) had itch‐dominant AD. Similar improvements in itch severity were observed after treatment with abrocitinib in patients with itch‐dominant AD as defined by baseline PP‐NRS and EASI score (Figure S1).

FIGURE 5Changes in itch and lesional severity over time as assessed by baseline PP‐NRS and IGA scores in patients treated with (a) abrocitinib 100 mg or (b) abrocitinib 200 mg (long‐term JADE EXTEND study). AD, atopic dermatitis; IGA, Investigator's Global Assessment; PP‐NRS, Peak Pruritus Numerical Rating Scale. ^a^Patients at study baseline with available data at both baseline and Week 24. ^b^Patients with available data at both Week 24 and Week 48. JADE EXTEND is an ongoing study and not all patients with Week 24 data had reached the Week 48 timepoint at the time of this analysis. No Itch = PP‐NRS 0 or 1; No Itch‐Moderate Itch = PP‐NRS 0−6; Mild‐Moderate Itch = PP‐NRS 2−6; Severe Itch = PP‐NRS score of 7−10; Clear‐Mild Lesions = IGA 0 or 1; Clear‐Moderate Lesions = IGA 0−3; Mild‐Moderate Lesions = IGA 2 or 3; Severe Lesions = IGA 4.

**Figure:**
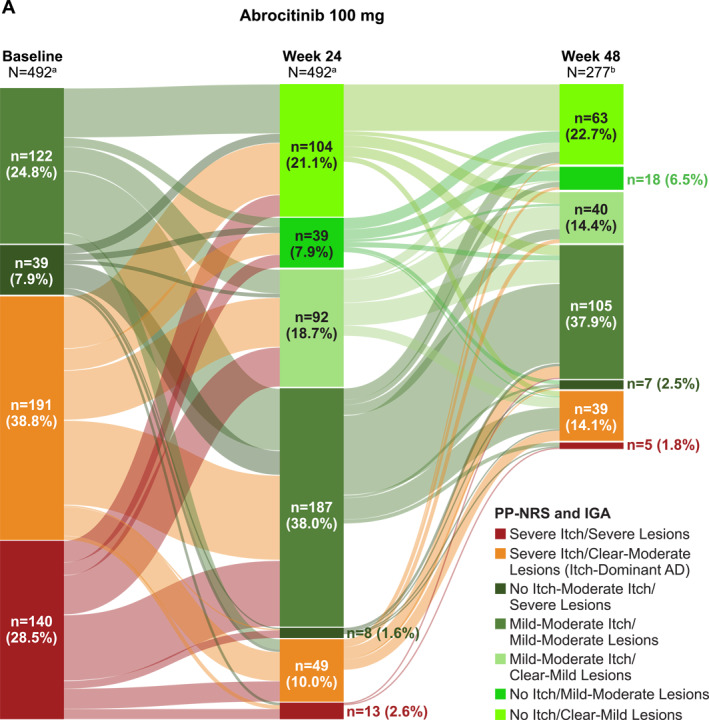

**Figure:**
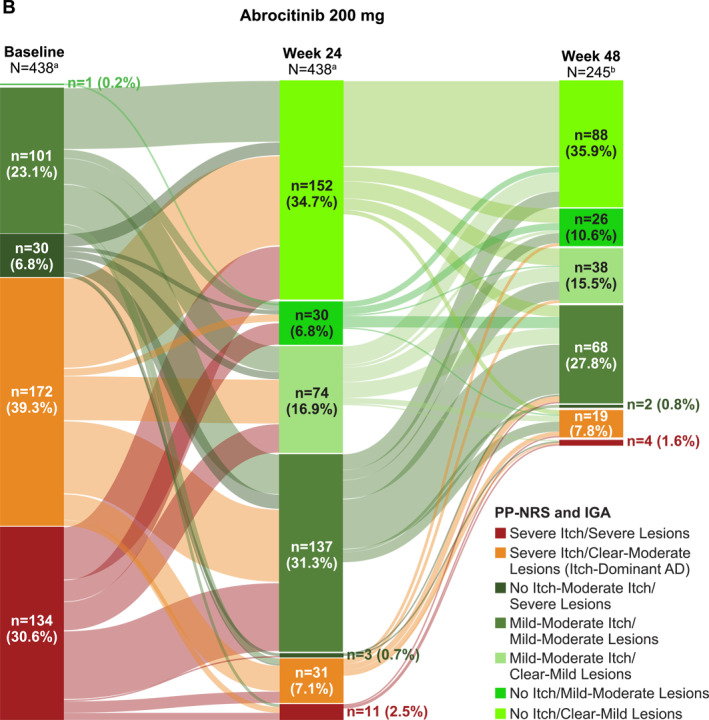

Most patients with itch‐dominant AD at baseline as assessed by PP‐NRS and IGA scores experienced itch improvement over time, with no itch‐moderate itch and clear‐moderate lesions observed in 86% of patients in the abrocitinib 100 mg group and 87% in the abrocitinib 200 mg group by Week 24 and 62% of patients in the abrocitinib 100 mg group and 67% of patients in the abrocitinib 200 mg group by Week 48 (Table S1). Moreover, at Week 24, 21% and 37% of patients with itch‐dominant AD at baseline treated with abrocitinib 100 mg and abrocitinib 200 mg, respectively, reported no itch or almost complete itch relief (ie, PP‐NRS score of 0 or 1) and were assessed as having clear (IGA 0) or almost clear (IGA 1) lesions (Table S1). Similar results were observed in patients with itch‐dominant AD at baseline as assessed by PP‐NRS and EASI scores (Table S2).

## 4 DISCUSSION

In this post hoc analysis of pooled data from the JADE clinical trial programme, more than one‐third (37%) of patients with moderate‐to‐severe AD had itch‐dominant AD at baseline defined by PP‐NRS and IGA scores, suggesting the pervasiveness of this clinical phenotype. At baseline, 57% of patients with itch‐dominant AD reported their disease as severe on the patient‐reported PtGA instrument; however, none of these patients were categorised as having severe disease on the physician‐reported IGA scale. This is in line with previous reports of disparities between patient‐ and physician‐reported assessments of AD severity and underscores the importance of considering both metrics for optimal treatment management in the clinical practice setting.^6^, ^21^

Following 12 weeks of treatment with abrocitinib 200 mg, 40% of patients with itch‐dominant AD per baseline PP‐NRS and IGA scores achieved a stringent threshold of itch improvement with a PP‐NRS score of <2, and almost half (45%) reported complete or almost complete clearance of AD symptoms based on the PtGA instrument. Similar findings were observed when itch‐dominant AD was defined according to baseline PP‐NRS and EASI scores. Abrocitinib efficacy in the itch‐dominant group was dose‐dependent and largely consistent with that observed in the overall population from abrocitinib monotherapy trials. Improvements in AD symptoms translated to better QoL outcomes; >26% of adults with itch‐dominant AD reported little to no impact of AD on their daily QoL after 12 weeks of treatment with abrocitinib at either dose. This is noteworthy given the high symptom burden and QoL impairment due to itch in patients with AD.^2^

Abrocitinib was also efficacious over the long term; most patients with itch‐dominant AD per baseline PP‐NRS and IGA scores experienced improvement, with no itch‐moderate itch and clear‐moderate lesions observed in 86%–87% of patients by Week 24 and 62%–67% of patients by Week 48. Furthermore, 37% of patients with itch‐dominant AD at baseline reported near to complete itch relief and lesion clearance at the stringent thresholds of PP‐NRS 0/1 and IGA 0/1 by Week 24 of treatment with abrocitinib 200 mg. This highlights the potential benefit of continued treatment with abrocitinib in patients with AD with a heterogenous presentation of symptoms and a high unmet need. The rapid and sustained itch response and robust efficacy observed with abrocitinib in this analysis is consistent with previous studies,^22^, ^23^ and may be attributable to the broad inhibition of multiple itch‐related cytokine pathways by JAK1 selective inhibitors.^24^, ^25^, ^26^

Therapeutic efficacy data are limited in patients with itch‐dominant AD. A phase 2 study evaluating the efficacy of the κ‐opioid receptor agonist difelikefalin in patients with itch‐dominant AD showed dose‐dependent improvements in itch at Week 12 compared with placebo.^27^ In a post hoc analysis of the phase 3 trial BREEZE‐AD7, patients with itch‐dominant AD treated with the JAK1/2 inhibitor baricitinib, had significantly greater improvements in itch and disease severity versus placebo at Week 16.^28^ To our knowledge, this is the first report of a systemic treatment showing long‐term efficacy up to 1 year in patients with AD who have the itch‐dominant phenotype.

The limitations of this analysis should be noted. The JADE monotherapy and COMPARE trials restricted the study population to those with moderate or severe AD and excluded patients with almost clear/mild skin lesions at baseline who may be characterised as having itch‐dominant AD. Therefore, the findings of this analysis may not be representative of the overall itch‐dominant population. It is unclear whether the patients included in this analysis had chronic itch‐dominant AD for the duration of their disease or if they were recruited to the study at a time in their disease trajectory where their symptoms were coincidentally categorised as itch‐dominant. Additionally, as JADE EXTEND is an ongoing study, not all patients had reached Week 48 at the time of analysis and thus the results shown represent an interim analysis as of the 22 April 2020 clinical cutoff. Lastly, this is a post hoc analysis of subgroups with relatively small sample sizes.

In this post hoc analysis, abrocitinib has shown rapid improvement in itch, including at stringent thresholds of improvement, in patients with itch‐dominant AD, for whom effective options are few. Improvements in AD symptoms translated to better QoL outcomes, and majority of patients with itch‐dominant AD at baseline experienced itch improvement without worsening of lesional severity by 48 weeks of abrocitinib treatment. Longer‐term outcomes with later data cutoffs will further characterise the efficacy profile of abrocitinib as a suitable treatment option for patients with itch‐dominant AD and inform its placement in the context of a rapidly changing therapeutic environment.

## CONFLICT OF INTEREST STATEMENT

J.I.S has served as an investigator for Celgene, Eli Lilly and Company, F. Hoffmann‐LaRoche, Menlo Therapeutics, Realm Therapeutics, Regeneron Pharmaceuticals, and Sanofi‐Genzyme, as a consultant for Pfizer Inc., AbbVie, Anacor, AnaptysBio, Arena Pharmaceuticals, Dermavant, Dermira, Eli Lilly and Company, Galderma, GlaxoSmithKline, Glenmark, Incyte, Kiniksa Pharmaceuticals, LEO Pharma, Menlo Therapeutics, Novartis, Realm Therapeutics, Regeneron Pharmaceuticals, and Sanofi‐Genzyme, and as a speaker for Regeneron Pharmaceuticals and Sanofi‐Genzyme. J.P.T is an employee of LEO pharma, and served as an advisor for Pfizer Inc., AbbVie, Almirall, Arena Pharmaceuticals, Aslan Pharmaceuticals, Coloplast, Eli Lilly and Company, LEO Pharma, OM Pharma, Union Therapeutics, Regeneron Pharmaceuticals, and Sanofi‐Genzyme, a speaker for Pfizer Inc., AbbVie, Almirall, Eli Lilly and Company, LEO Pharma, Regeneron Pharmaceuticals, and Sanofi‐Genzyme, and received research grants from Pfizer Inc., Regeneron Pharmaceuticals, and Sanofi‐Genzyme. I.L is an employee of IQVIA, who were paid contractors to Pfizer Inc. in the development of this manuscript and in providing statistical support. D.E.M, and E.G are employees and shareholders of Pfizer Inc. R.C has served as an advisory board member, consultant, and/or investigator for Pfizer Inc., AbbVie, Apogee, Arcutis, Arena, Argenx, ASLAN, Beiersdorf, Boehringer Ingelheim, Bristol Myers Squibb, Cara Therapeutics, Dermavant, Eli Lilly and Company, EPI Health, Incyte, LEO Pharma, L’Oréal, National Eczema Association, Regeneron Pharmaceuticals, Sanofi‐Genzyme, and UCB, and as a speaker for Pfizer Inc., AbbVie, Arcutis, Beiersdorf, Dermavant, Eli Lilly and Company, EPI Health, Incyte, LEO Pharma, Regeneron Pharmaceuticals, Sanofi‐Genzyme, and UCB.

## AUTHOR CONTRIBUTIONS

**Jonathan I. Silverberg**: Conceptualization, formal analysis, methodology, writing – review & editing. **Jacob P. Thyssen**: Conceptualization, formal analysis, methodology, writing – review & editing. **Irina Lazariciu**: Conceptualization, data curation, formal analysis, investigation, writing – review & editing. **Daniela Myers**: Conceptualization, methodology, writing – review & editing. **Erman Güler**: Conceptualization, methodology, writing – review & editing. **Raj Chovatiya**: Conceptualization, methodology, writing – review & editing.

## ETHICS STATEMENT

All evaluated studies in this analysis were approved by institutional review boards or ethics committees at each site. All patients provided written informed consent.

## Supporting information

Supporting Information S1

## Data Availability

Upon request, and subject to review, Pfizer will provide the data that support the findings of this study. Subject to certain criteria, conditions and exceptions, Pfizer may also provide access to the related individual de‐identified participant data. See https://www.pfizer.com/science/clinical‐trials/trial‐data‐and‐results for more information.
