# Supplementary material for: Abrocitinib may improve itch and quality of life in patients with itch‐dominant atopic dermatitis
Source: Skin Health Dis. 2024 May 5;4(4):e382. doi: 10.1002/ski2.382 (PMC11297436; doi:10.1002/ski2.382)
Supplement: Supplementary file 1 — Supporting Information S1 [file SKI2-4-e382-s001.docx]

**SUPPLEMENTARY MATERIAL**

**Abrocitinib may improve itch and quality of life in patients with itch-dominant atopic dermatitis**

Jonathan I. Silverberg,^1^ Jacob P. Thyssen,^2*^ Irina Lazariciu,^3^ Daniela E. Myers,^4^ Erman Güler,^5^ Raj Chovatiya^6^

^1^Department of Dermatology, The George Washington University School of Medicine and Health Sciences, Washington, DC, USA; ^2^Department of Dermatology, Bispebjerg Hospital, University of Copenhagen, Copenhagen, Denmark; ^3^Department of Biostatistics, IQVIA, Kirkland, Quebec, Canada; ^4^Pfizer Inc., Collegeville, PA, USA; ^5^Pfizer Inc., Istanbul, Turkey; ^6^Department of Dermatology, Northwestern University Feinberg School of Medicine, Chicago, IL, USA

*Affiliation at the time of the study

**Supplementary Tables and Figures**

**Supplementary Table S1**. Changes in itch and lesional severity over time in patients with itch-dominant AD by baseline PP-NRS and IGA scores (Long-term JADE EXTEND study)

|  | | Itch improvement without worsening of lesional severity,  n (%) | | | | | Itch improvement with worsening of lesional severity or no itch improvement with or without worsening of lesional severity, n (%) | | | |
| --- | --- | --- | --- | --- | --- | --- | --- | --- | --- | --- |
|  |  | No Itch/Clear Lesions | No Itch/Mild-Moderate Lesions | Mild-Moderate Itch/Clear Lesions | Mild-Moderate Itch/Mild-Moderate Lesions | Total | No Itch-Moderate Itch/Severe Lesions | Severe Itch/Clear-Moderate Lesions | Severe Itch/Severe Lesions | Total |
| **Baseline to Week 24**^a^ | Abrocitinib  100 mg  n=191 | 41 (21.5) | 17 (8.9) | 39 (20.4) | 67 (35.1) | 164 (85.9) | 1 (0.5) | 23 (12.0) | 3 (1.6) | 27 (14.1) |
|  | Abrocitinib  200 mg  n=172 | 63 (36.6) | 5 (2.9) | 31 (18.0) | 51 (29.7) | 150 (87.2) | 0 | 18 (10.5) | 4 (2.3) | 22 (12.8) |
| **Week 24 to Week 48**^b^ | Abrocitinib  100 mg  n=24 | 1 (4.2) | 2 (8.3) | 3 (12.5) | 9 (37.5) | 15 (62.5) | 1 (4.2) | 8 (33.3) | 0 | 9 (37.5) |
|  | Abrocitinib  200 mg  n=12 | 0 | 2 (16.7) | 2 (16.7) | 4 (33.3) | 8 (66.7) | 0 | 3 (25.0) | 1 (8.3) | 4 (33.3) |

AD, atopic dermatitis; IGA, Investigator’s Global Assessment; PP-NRS, Peak Pruritus Numerical Rating Scale.

^a^Patients with itch-dominant AD at study baseline with available data at both baseline and week 24.

^b^Patients with itch-dominant AD at week 24 with available data at both week 24 and week 48. JADE EXTEND is an ongoing study and not all patients with week 24 data had reached the week 48 timepoint at the time of this analysis.

No itch = PP-NRS 0/1; No itch-Moderate Itch = PP-NRS 0−6; Mild-Moderate itch = PP-NRS 2−6; Severe itch = PP-NRS 7−10; Clear-Mild lesions = IGA 0 or 1; Clear-Moderate lesions = IGA 0-3; Mild-Moderate lesions, IGA 2 or 3; Severe lesions = IGA 4.

**Supplementary Table S2**. Changes in itch and lesional severity over time in patients with itch-dominant AD by PP-NRS and EASI scores (Long-term JADE EXTEND study)

|  | | Itch improvement without worsening of lesional severity, n (%) | | | | | Itch improvement with worsening of lesional severity or no itch improvement with or without worsening of lesional severity, n (%) | | | |
| --- | --- | --- | --- | --- | --- | --- | --- | --- | --- | --- |
|  |  | No Itch/Clear Lesions | No Itch/Mild-Moderate Lesions | Mild-Moderate Itch/Clear Lesions | Mild-Moderate Itch/Mild-Moderate Lesions | Total | No Itch-Moderate Itch/Severe Lesions | Severe Itch/Clear-Moderate Lesions | Severe Itch/Severe Lesions | Total |
| **Baseline to Week 24**^a^ | Abrocitinib  100 mg  n=96 | 25 (26.0) | 0 | 44 (45.8) | 12 (12.5) | 81 (84.4) | 1 (1.0) | 11 (11.5) | 3 (3.1) | 15 (15.6) |
|  | Abrocitinib  200 mg  n=79 | 30 (38.0) | 1 (1.3) | 30 (38.0) | 9 (11.4) | 70 (88.6) | 0 | 7 (8.9) | 2 (2.5) | 9 (11.4) |
| **Week 24 to Week 48**^b^ | Abrocitinib  100 mg  n=23 | 2 (8.7) | 1 (4.3) | 7 (30.4) | 3 (13.0) | 13 (56.5) | 2 (8.7) | 8 (34.8) | 0 | 10 (43.5) |
|  | Abrocitinib  200 mg  n=14 | 2 (14.3) | 0 | 3 (21.4) | 4 (28.6) | 9 (64.3) | 0 | 4 (28.6) | 1 (7.1) | 5 (35.7) |

AD, atopic dermatitis; EASI, Eczema Area and Severity Index; PP-NRS, Peak Pruritus Numerical Rating Scale.

^a^Patients with itch-dominant AD at study baseline with available data at both baseline and week 24.

^b^Patients with itch-dominant AD at week 24 with available data at both week 24 and week 48. JADE EXTEND is an ongoing study and not all patients with week 24 data had reached the week 48 timepoint at the time of this analysis.

No itch = PP-NRS 0/1; No itch-Moderate Itch = PP-NRS 0−6; Mild-Moderate itch = PP-NRS 2−6; Severe itch = PP-NRS 7−10; Clear-Mild lesions = EASI 0−6; Clear-Moderate lesions = EASI 0−21; Mild-Moderate lesions = EASI 6−21; Severe lesions = EASI >21.

**Supplementary Figure S1.** Changes in itch and lesional severity over time as assessed by PP-NRS and EASI scores in patients treated with (**A**) abrocitinib 100 mg or (**B**) abrocitinib 200 mg (Long-term JADE EXTEND study)


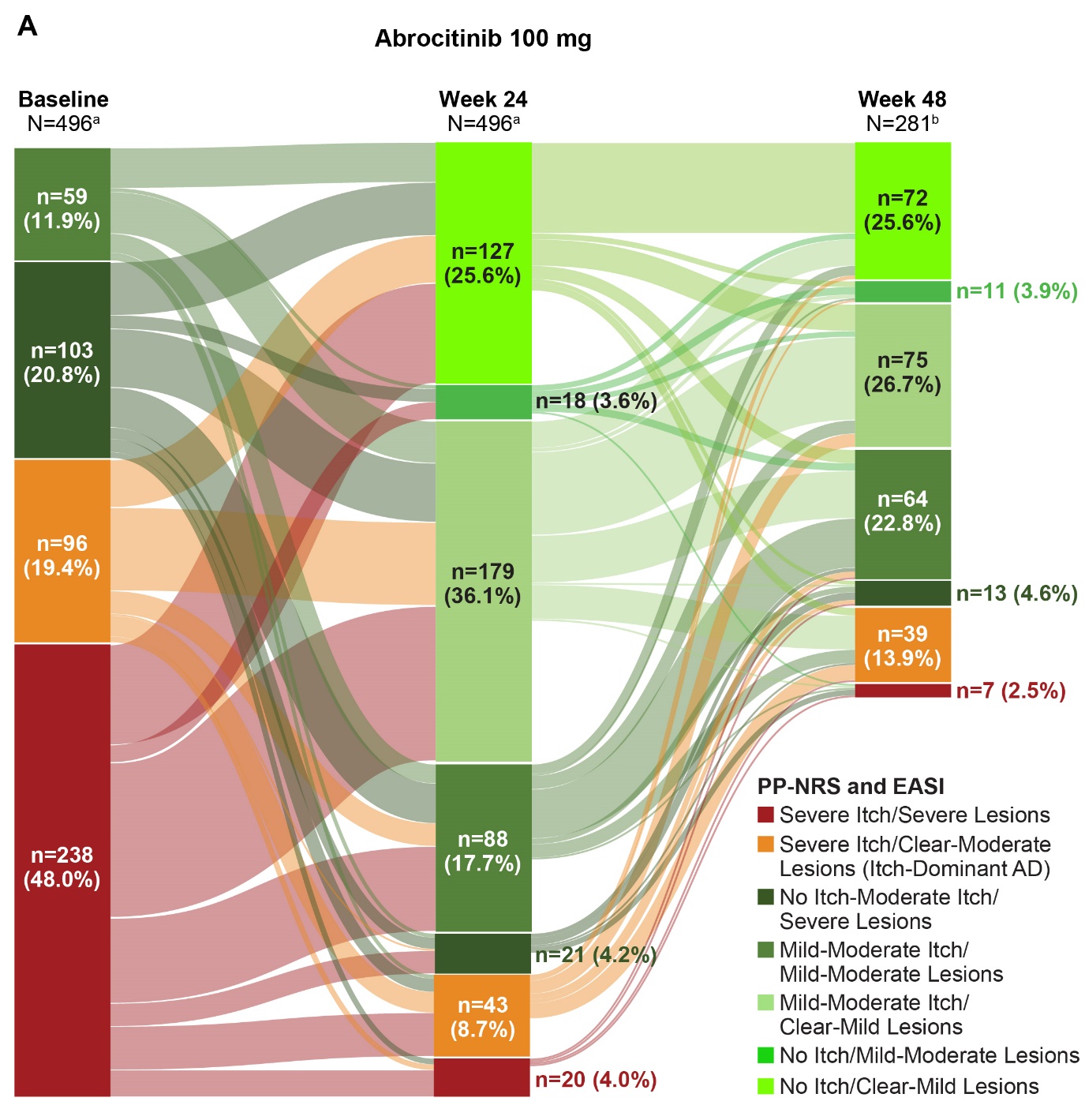


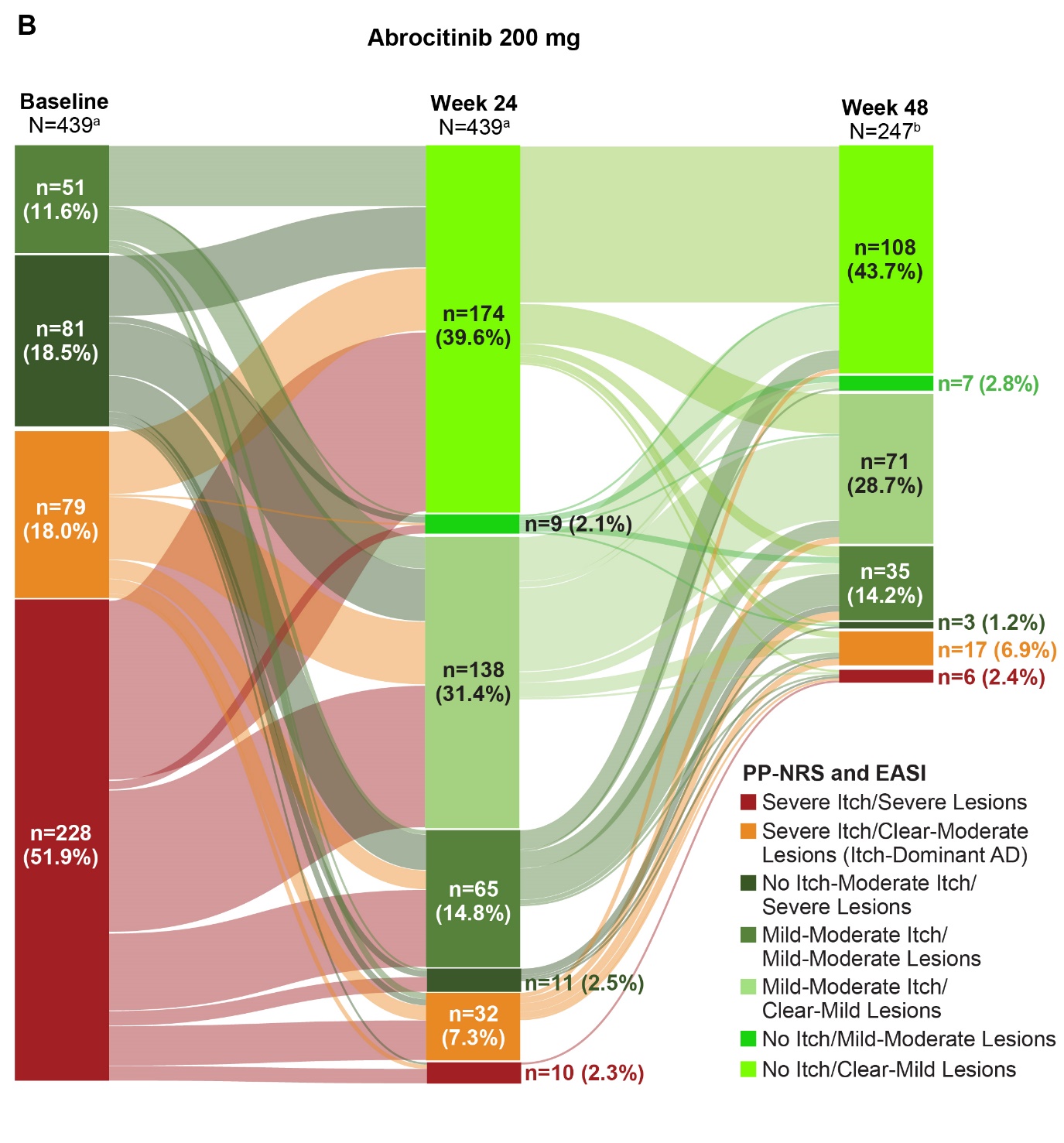


AD, atopic dermatitis; EASI, Eczema Area and Severity Index; PP-NRS, Peak Pruritus Numerical Rating Scale.

^a^Patients at study baseline with available data at both baseline and week 24.

^b^Patients with available data at both week 24 and week 48. JADE EXTEND is an ongoing study and not all patients with week 24 data had reached the week 48 timepoint at the time of this analysis.

No itch = PP-NRS 0/1; No itch-Moderate Itch = PP-NRS 0−6; Mild-Moderate itch = PP-NRS 2−6; Severe itch = PP-NRS 7−10; Clear-Mild lesions = EASI 0−6; Clear-Moderate lesions = EASI 0−21; Mild-Moderate lesions = EASI 6−21; Severe lesions = EASI >21.
